# PatWRKY71 transcription factor regulates patchoulol biosynthesis and plant defense response

**DOI:** 10.1186/s12870-023-04660-7

**Published:** 2024-01-02

**Authors:** Jian Li, Huan-Chao Huang, Yue-Qiu Zuo, Ming-Yong Zhang, Meng-Ling He, Kuai-Fei Xia

**Affiliations:** 1 State Key Laboratory of Plant Diversity and Specialty Crops, South China Botanical Garden, Chinese Academy of Sciences, Guangzhou, 510650 China; 2 South China Botanical Garden, Chinese Academy of Sciences, Guangzhou, 510650 China; 3 School of Traditional Chinese Medicine, Guangdong Pharmaceutical University, Guangzhou, 510006 China

**Keywords:** Pogostemon cablin, Patchoulol biosynthesis, Plant defense response, *PatWRKY71*, Transcription factor

## Abstract

Patchoulol, a valuable compound belonging to the sesquiterpenoid family, is the primary component of patchouli oil produced by *Pogostemon cablin* (*P. cablin*). It has a variety of pharmacological and biological activities and is widely used in the medical and cosmetic industries. However, despite its significance, there is a lack of research on the transcriptional modulation of patchoulol biosynthesis.

Salicylic acid (SA), is a vital plant hormone that serves as a critical signal molecule and plays an essential role in plant growth and defense. However, to date, no studies have explored the modulation of patchoulol biosynthesis by SA. In our study, we discovered that the application of SA can enhance the production of patchoulol. Utilizing transcriptome analysis of SA-treated *P. cablin*, we identified a crucial downstream transcription factor, PatWRKY71. The transcription level of *PatWRKY71* was significantly increased with the use of SA. Furthermore, our research has revealed that PatWRKY71 was capable of binding to the promoter of *PatPTS*, ultimately leading to an increase in its expression. When *PatWRKY71* was silenced by a virus, the expression of both *PatWRKY71* and *PatPTS* was reduced, resulting in the down-regulation of patchoulol production. Through our studies, we discovered that heterologous expression of *PatWRKY71* leads to an increase in the sensitivity of *Arabidopsis* to salt and Cd, as well as an outbreak of reactive oxygen species (ROS). Additionally, we uncovered the regulatory role of *PatWRKY71* in both patchoulol biosynthesis and plant defense response. This discovery provided a theoretical basis for the improvement of the content of patchoulol and the resistance of *P. cablin* through genetic engineering.

**Supplementary Information:**

The online version contains supplementary material available at 10.1186/s12870-023-04660-7.

## Introduction

Patchouli, scientifically known as *P. cablin* (Blanco) Benth, is a vital component of traditional Chinese medicine. It is a perennial herb belonging to the Lamiaceae family, known for its aromatic properties and versatility. The dried aerial parts of patchouli possess potent medicinal properties, making it beneficial in several health conditions such as antibacterial, anti-inflammatory, analgesic, anti-gastric ulcer, and anti-photoaging, among others [1]. Patchoulol is a sesquiterpene and the primary element found in patchouli oil, which is produced by the patchouli plant and holds great economic significance [2]. Despite this, the availability of both patchouli oil and patchoulol is considerably limited in the market.

In plants, the synthesis of sesquiterpenoids involves two main pathways. The first pathway, which takes place in the cytoplasm, is the mevalonic acid (MVA) pathway that utilizes acetyl-CoA as the substrate. The second pathway, the methylerythritol 4-phosphate (MEP) pathway, occurs in the plastid and uses pyruvate and glyceraldehyde 3-phosphate as substrates. The synthesis of patchoulol, an important sesquiterpenoid, is catalyzed by patchoulol synthase (*PatPTS*), which is a critical enzyme in the rate-limiting step that converts farnesyl pyrophosphate into patchoulol. Studies revealed that transcription factors (TFs) have crucial regulatory functions in terpenoid biosynthesis [3]. The WRKY transcription factor AaGSW1 has proven to be particularly effective in enhancing artemisinin and dihydroartemisinic acid contents by binding directly to the W-box motifs in the promoters of *CYP71AV1* and *AaORA*. In addition, *AaGSW1* is subject to direct regulation by AaMYC2 and AabZIP1 [4]. AaWRKY9 bound to the promoters of *AaGSW1* or *AaDBR2*, positively regulating their expression and resulting in the promotion of artemisinin biosynthesis [5]. Meanwhile, HcMYB proteins directly bound to the promoters of bottom structural volatile synthesis genes in *H. coronarium*, which are associated with the regulation of floral volatile contents [6]. As R2R3-MYB transcription factors, HcMYB1 and HcMYB2 interacted with the *HcBSMT2* promoter and significantly affected the levels of floral scent compounds [7]. According to some studies, WRKY transcription factors have also been discovered to regulate the synthesis of terpenoid metabolites in *Lamiaceae* plants. For example, in *Salvia miltiorrhiza*, SmWRKY34 has been shown to have a negative effect on phenolic acids and tanshinones by directly regulating *SmRAS* and *SmGGPPS* [8]. Similarly, overexpression of *SmWRKY2* in *S. miltiorrhiza* hairy roots has been found to significantly enhance the expression of *SmDXS2* and *SmCPS*, resulting in an increased accumulation of tanshinones [9].

Currently, studies were underway to investigate the transcription factors that regulate patchoulol biosynthesis in *P. cablin*. One such study found that PcWRKY44 bound to the promoter of *PcFPPS* and up-regulated the expression levels of both *PcFPPS* and *PcPTS*, effectively promoting patchouli alcohol biosynthesis [10]. Basic leucine zipper (bZIP) transcription factor PcbZIP44 bound to the promoter of *PcPTS* and repressed its expression, resulting in lower levels of patchoulol [11].

As a vital plant hormone, salicylic acid (SA) served as a significant signal molecule and plays a critical role in defending against both abiotic and biotic stresses in plants [12]. Research had demonstrated that SA can enhance plant tolerance to salt, heat, drought, heavy metals, and disease [13]. Additionally, recent studies indicated SA’s ability to regulate plant secondary metabolism. The subject study suggested that SA can increase the activity of phenylalanine ammonia-lyase (PAL), which in turn, results in a rise in the levels of total soluble phenolics and phenolic acids [14]. Furthermore, SA had the potential to stimulate Taxol production in *Taxus baccata* [15]. Despite these findings, it remains unclear whether SA treatment can influence patchoulol production in *P. cablin*.

In the course of our study, we discovered that SA has the potential to enhance patchoulol production. Additionally, we utilized transcriptome analysis of SA-treated *P. cablin* to identify a transcription factor known as *PatWRKY71*. We observed an up-regulation in the transcription level of *PatWRKY71* due to SA treatment. Furthermore, we found that PatWRKY71 binds to the promoter of *PatPTS*, boosting its expression. Interestingly, our experiments also showed that silencing of *PatWRKY71* leads to a decrease in both its own expression and that of *PatPTS*, resulting in a decline in patchoulol biosynthesis. The heterologous expression of *PatWRKY71* had been found to increase salt sensitivity and Cd sensitivity in *Arabidopsis*, which was accompanied by a rise in ROS outbreak. Our studies have revealed the regulatory role of *PatWRKY71* in both patchoulol biosynthesis and plant defense response. This discovery can provide a theoretical basis for improving patchoulol content and enhancing *P. cablin* resistance through genetic engineering.

## Materials and methods

### Plant materials

The *P. cablin* plants were collected from School of Traditional Chinese Medicine, Guangdong Pharmaceutical University, Guangzhou City, Guangdong Province, China. *P. cablin* plants at 6–8-leaf stage were used in experiments.

### Transcriptome analysis

The leaves, both with and without SA treatment, were collected and RNA was extracted using the TRlzol Reagent instruction manual (Life Technologies, California, USA). The samples were sequenced using the Illumina HiSeq™ sequencing platform. Low quality reads, such as only adaptor, unknown nucleotides > 5%, or Q20 < 20% (percentage of sequences with sequencing error rates < 1%), were removed by perl script. The resulting clean reads were filtered and mapped to the patchouli reference genome [16] using TopHat2 software [17]. The aligned records from the aligners in BAM/SAM format were further examined to remove potential duplicate molecules. The gene expression levels were estimated using FPKM values, which stands for fragments per kilobase of exon per million fragments mapped. This was done using the Cufflinks software developed by Trapnell et al. in 2012 [18]. For subsequent analysis, only genes with an absolute value of log2 ratio greater than or equal to two and an False Discovery Rate (FDR) significance score of less than or equal to 0.01 were used. To perform gene ontology analysis, we utilized Cluster Profiler in R software codes, as developed by Yu et al. in 2012 [19]. The pheatmap package was utilized to conduct differential expression analyses via bidirectional hierarchical clustering in R. The package can be found at https://cran.r-project.org/web/packages/pheatmap/index.html.

### Patchoulol extraction and analysis

To determine patchoulol levels, leaf discs of *P. cablin* were obtained using a 6 mm diameter hole punch. Samples were collected using headspace sampling (HE) and solid phase microextraction (SPME), and analyzed using an Agilent 8890 Gas Chromatograph coupled with a 5977B MSD (Mass Selective Detector) from Agilent in the United States. In brief, the samples were added to a 15 mL headspace bottle and incubated for 10 min at 50 ℃. The extraction was carried out using a Divinylbenzene/Carboxen/Polydimethylsiloxane (DVB/CAR/PDMS) 50/30 μm StableFlex Fiber at 50 ℃ for 5 min, followed by desorption at 250 ℃ for 1 min. The analysis was performed using an HP-5 ms capillary column (0.25 mm × 30 m × 0.25 μm, Agilent). The separation conditions for the subject paper were as follows: the initial column temperature was set to 45℃ for 0.5 min. It was then increased by 10℃/min until it reached 280℃, where it was maintained for 0.5 min. The ion source used was EI (Electron Ionization), and the carrier gas was High Purity Helium (99.999%) at a flow rate of 1.2 mL per minute. The transfer line temperature was set at 250 °C, and ions were generated by a 70 eV electron beam. Full-scan mode was utilized to scan the mass range from m/z 50 to 450 Da. Metabolites were identified by leveraging the National Institute of Standard and Technologies (NIST) online library (https://webbook.nist.gov/chemistry/cas-ser.html) through the MassHunter software (Agilent Technologies).

### Subcellular localization of PatWRKY71

To determine the subcellular localization of PatWRKY71, we amplified its sequence and inserted it into the pFGC binary vector, which contains the UBQ10 promoter. This allowed us to obtain the *pFGC-UBQ10: PatWRKY71* vector. To further investigate its localization, we co-expressed *UBQ10: GFP: PatWRKY71* and a nuclear marker in protoplasts isolated from *Arabidopsis* leaves [20]. After 16 h of culturing, we observed the transfected protoplasts using confocal microscopy (TCS SP8; Leica). To observe GFP, we set the excitation/emission wavelength at 488 nm/500 to 530 nm. For mCherry, we used an excitation/emission wavelength of 561 nm/580 to 630 nm. Finally, to observe chlorophyll, we set the excitation/emission wavelength at 548 nm/650 to 750 nm.

### Virus-induced gene silencing (VIGS) assay

The method of VIGS was carried out as previously described [21] to silence the expression of *PatWRKY71*. The 483 bp CDS fragment of *PatWRKY71* was cloned into the pTRV2 vector to obtain *TRV-PatWRKY71*. The Agrobacterium EHA105 suspension containing the target vectors was then injected into the leaves of *P. cablin*. After 14 days, the leaves were collected for RT-qPCR to measure the expression level of target genes and for GC–MS analysis to measure patchoulol.

### Yeast one-hybrid (Y1H)

Y1H assay was conducted following the methodology described in Liu et al. [22]. The promoter sequence of *PatPTS*, which spans approximately 1.5 k bp upstream of the transcription starting site, was cloned into the multiple cloning site of the pBait-AbAi vector. *PatWRKY71* was cloned into the pGADT7 vector. The process of yeast transformation and confirmation was conducted in accordance with the instructions provided in the user manual of the Matchmaker Gold Yeast one-hybrid system. After selecting positively transformed yeasts, they were subjected to testing on SD/-Leu medium that contained 200 ng/ml Aureobasidin A (AbAi). As positive controls, p53-pAbAi and p53-pGADT7 were utilized. The primers used in the Y1H assay were listed in Supplemental Table 1.

### Dual luciferase(dual-LUC) reporter assay

The Dual-LUC assay was conducted according to the method described in Gao et al. [23]. To create the LUC reporter plasmid, the promoter of *PatPTS* was cloned into pGreenII 0800-LUC. The effector plasmid, pCAMBIA1300-GFP, was used to insert the ORF of *PatWRKY71*. The reporter and effector plasmids were transformed into separate *Agrobacterium tumefaciens* strains, GV3101, and incubated at 28℃ for 2–3 days. Agrobacterium culture was prepared and injected into *N. benthamiana* leaves. To measure LUC activity, we utilized the Dual-LUC Reporter Assay System (E2920; Promega, Madison, WI, USA) and normalized it to REN activity. The primers employed in the dual-LUC assay can be found in Supplemental Table 1.

### Plate assay

In the salt-sensitivity experiment, we sowed surface-sterilized seeds on 1/2 × MS medium with or without 100 μM NaCl. The seeds were then grown horizontally in a greenhouse at 22 °C under a 16-h light/8-h dark cycle. After two weeks of incubation, we recorded photos to document the results. For the Cd-sensitivity experiment, we surface-sterilized seeds and sowed them on 1/2 × MS medium containing 25 μM CdCl_2_. The seeds were grown horizontally for three weeks and we recorded their phenotype to determine the effects of cadmium on their growth. Furthermore, after being grown vertically on 1/2 × MS for 7 days, the seedlings were transferred to a solution of 1/2 × MS containing 25 μM CdCl_2_. The length of the roots was then measured.

### Histochemical assays

In order to detect H_2_O_2_, we placed detached leaves into a 1 mg/ml DAB solution and vacuumed them for two minutes. Following this, the samples were incubated in the dark for eight hours and then de-stained using a de-staining solution consisting of acetic acid, glycerol, and ethanol in a 1:1:3 ratio (v/v/v). In order to observe O_2_^.−^, we placed detached leaves in a pretreated buffer consisting of 10 mM NaN_3_ and 10 mM K_2_HPO_4_ at a pH of 7.8. We then vacuumed them for 30 s. After incubating the leaves in the pretreated buffer for 30 min, we transferred them to a 0.1% NBT solution and incubated them for 20 min in the dark. Finally, we de-stained the samples using a de-staining solution before observation.

### Quantitative RT-PCR analysis

The RNAprep Pure Plant Kit (DP432, TIANGEN) was used to extract total RNA, following the guidelines provided in the manual. Subsequently, cDNA was obtained through reverse transcription using the PrimeScript RT reagent Kit (DRR047A, TAKARA). To perform real-time PCR, the SYBR Premix ExTaq kit (Takara, RR820L) was used in accordance with the manufacturer's instructions, with the LightCycler480 (Roche) serving as the platform for the analysis. 2^−△△CT^ method was used to measure the target genes’ relative expression [24]. The primers used in quantitative RT-PCR are listed in Supplemental Table 1.

## Results

### SA-induced enhancement of patchoulol production in *P. cablin*

**Fig. 1:**
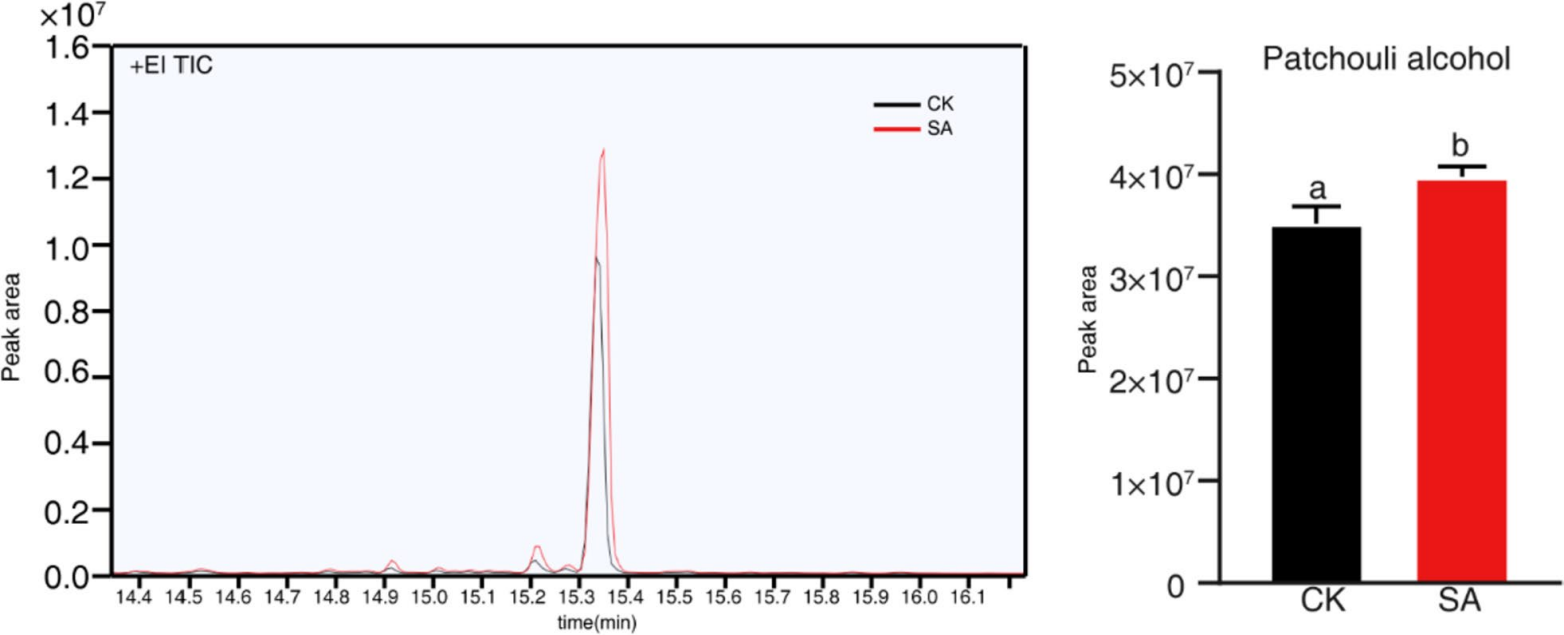
SA induce patchoulol production in *P. cablin*. *P. cablin* leaves at the 6–8 leaf stage were sprayed with 50 μM SA and measured the patchoulol levels after 7 days using HE-SPME–GC–MS, following the methods described in Methods. CK was defined as ethanol. Ethanol was used as solvent control and Student's t-test (*p* < 0.05) was utilized to determine significant differences from the control group, represented by different letters. Two similar results were obtained from independent samples

In order to examine the impact of SA on patchoulol biosynthesis, we administered a spray of 50 μM SA onto the leaves of *P. cablin*. After 7 days, we measured the levels of patchoulol using GC–MS. The results, displayed in Fig. 1, indicated that the SA-treated group displayed a significantly higher amount of patchoulol than the CK-group.

### Transcriptome analysis of *P. cablin* treated with SA

**Fig. 2:**
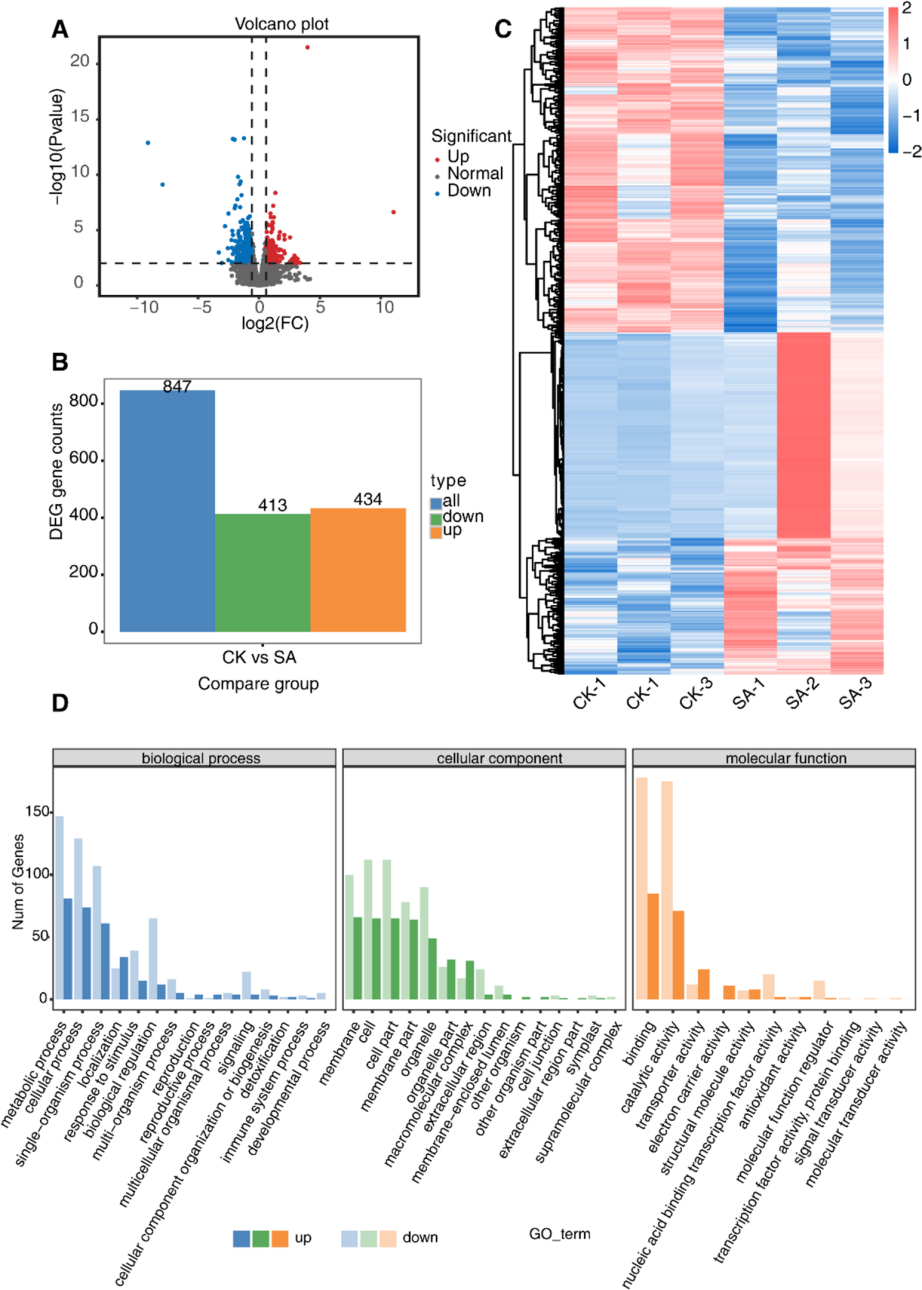
Differential analysis of gene expression through RNA-Sequencing. **A** Volcano plot analysis of the gene expression in the samples with or without 50 μM SA treatment. Abscissa represent log2 (fold change), ordinate represent -log 10 (adjusted P-value). **B** Differential expression gene analysis (DEGs) with or without SA treatment. 413 genes were up-regulated and 434 genes were down-regulated in SA-treated group compared with control group. **C** Hierarchical clustering analysis of SA—induced differentially expressed genes. **D** Gene Ontology categories of significantly down-regulated and up-regulated genes in *P. cablin*

**Fig. 3:**
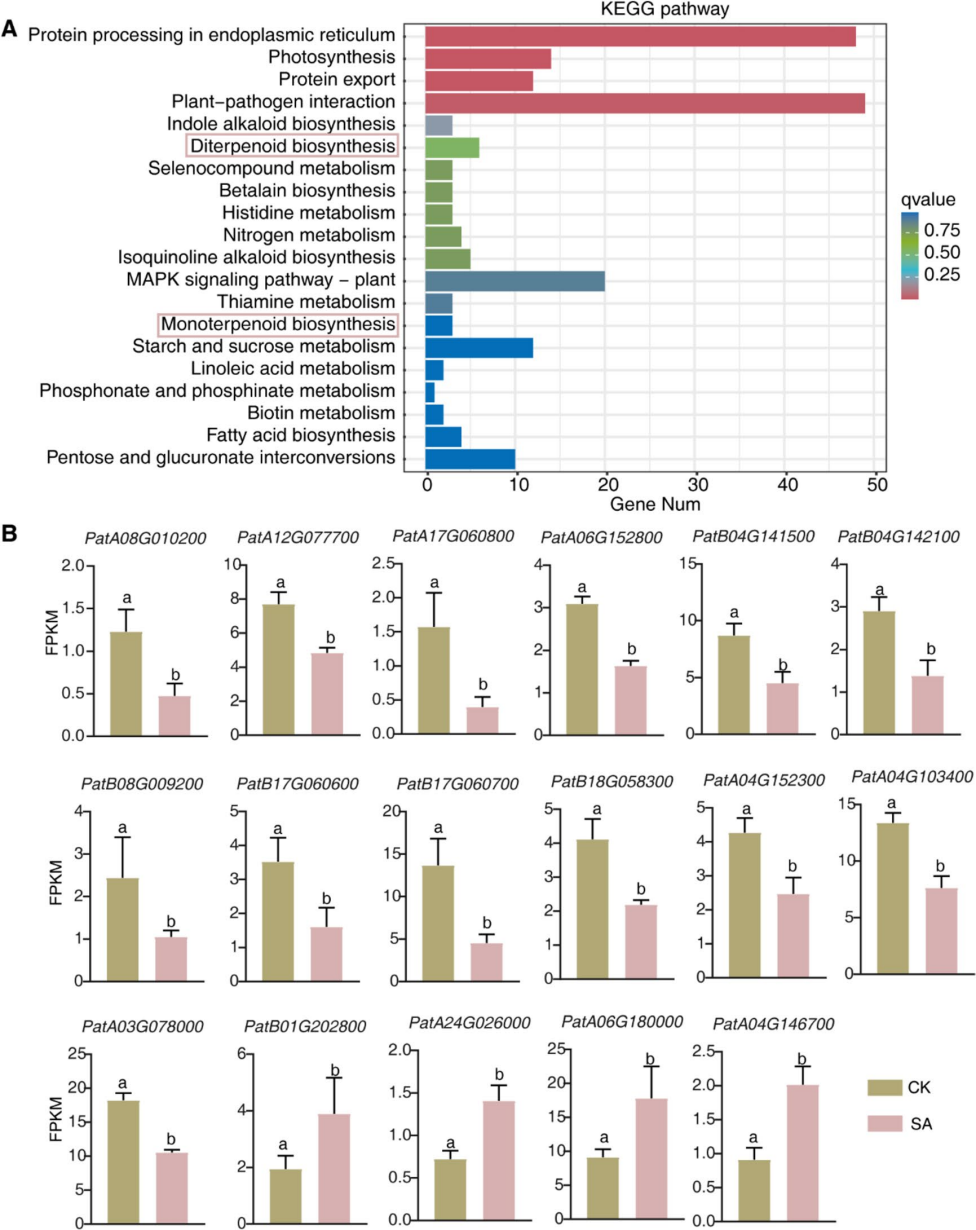
SA regulated the expression of genes related to terpenoid biosynthesis. **A** KEGG pathway analysis of differential expression genes in SA-treated samples. **B** FPKM of genes related with terpenoid biosynthesis. in transcriptome of *P. cablin* treated with SA

In order to investigate the reason for promoting patchoulol accumulation in plants treated with SA, we conducted an analysis of the global gene expression changes in these plants using RNA sequencing. Our analysis revealed 847 genes that exhibited a fold change ≥ 2 and significant differential expression between the SA-treated and control-treated plants (with an FDR ≤ 0.01). Out of these 847 genes, 434 genes were up-regulated while 413 genes were down-regulated (Fig. 2A-C). The genes that were differentially expressed underwent gene ontology analyses and were sorted into categories of biological process (BP), cellular component (CC), and molecular function (MF). Within the SA-treated pool, enriched BP included metabolic process, localization, and response to stimulus. The enriched CCs after SA treatment were membrane, macromolecular complex, and membrane-enclosed lumen. In the subject paper, it was found that MF such as "binding," "catalytic activity," and "transporter activity" were enriched in samples after SA spraying (Fig. 2D). Furthermore, KEGG analyses revealed that differentially expressed genes were significantly enriched in functions related to protein processing in the endoplasmic reticulum, plant-pathogen interaction, and photosynthesis, among others (Fig. 3A). Furthermore, it was observed that the differentially expressed genes (DEGs) in the terpenoid biosynthesis pathways underwent significant changes. Specifically, in the genes linked to terpenoid synthesis, 13 were notably down-regulated, while 3 were up-regulated in samples treated with SA (Fig. 3B). This indicated that the expression of genes involved in terpenoid biosynthesis was influenced by SA, which ultimately promoted the accumulation of patchoulol.

### The transcription factor *PatWRKY71* was found to be up-regulated in response to SA induction

**Fig. 4:**
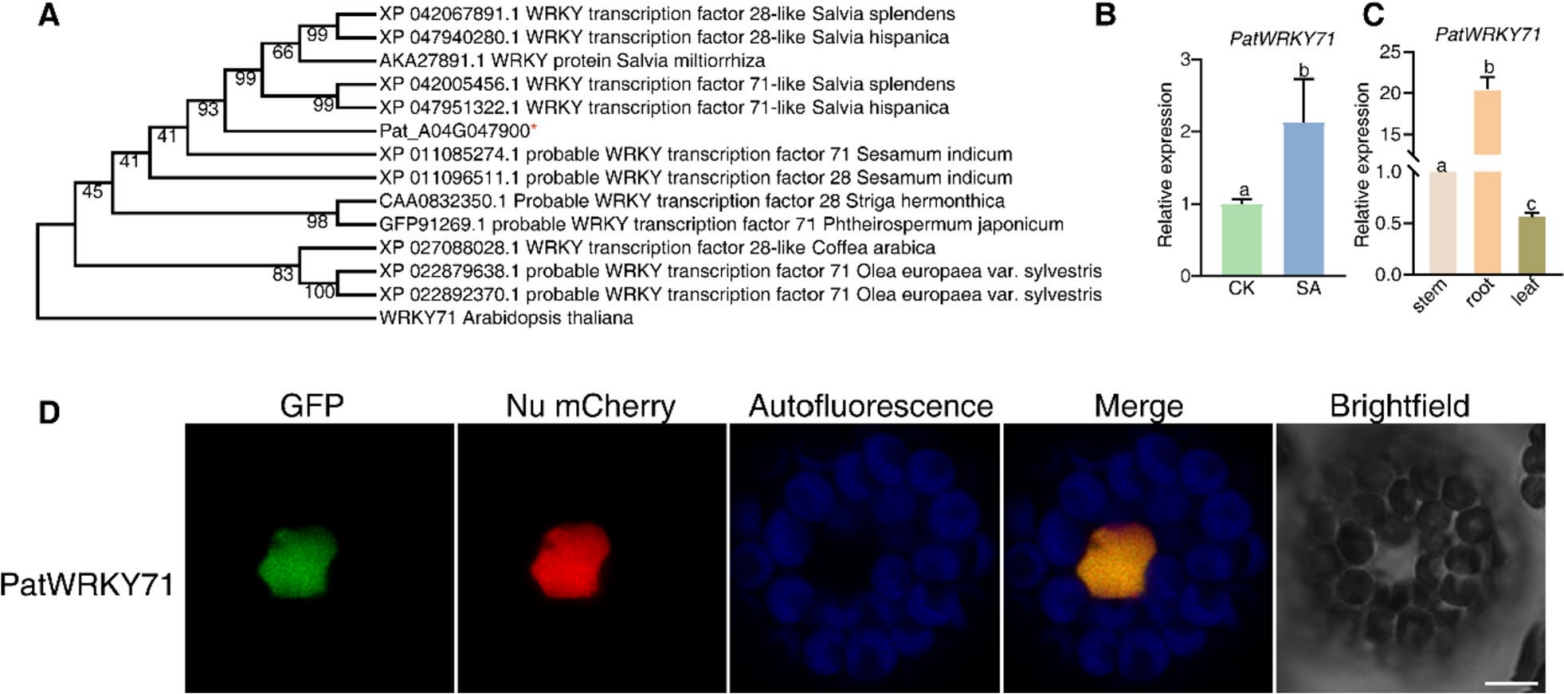
*PatWRKY71* induced by SA in *P. cablin*. **A** Phylogenetic trees of *PatWRKY71* between species. **B** Measure the relative expression of *PatWRKY71* by qRT-PCR. *P. cablin* leaves at 6–8-leaf stage were sprayed with 50 μM SA and leaves were collected after spaying 24 h. The internal control was expression level of *Pat18S.* Different letters represent a significant difference from the control-group using Student’s *t*-test (*p* < 0.05). Error bars indicate SE. **C** Detection of expression of *PatWRKY71* in different tissues. The internal control was expression level of *Pat18S*. Gene expression values are normalized to the stem average level, which was set as 1. Different letters represent a significant difference from the stem average level using Student’s *t*-test (*p* < 0.05). Error bars indicate SE. **D** Subcellular localization of PatWRKY71. The fusion plasmid *UBQ10: GFP: PatWRKY71* was co-expressed with the Nuclear marker (Nu) mCherry marker by transient expression in protoplasts. After 16 h incubation, images were recorded by confocal microscopy. Bar = 5 μm

To expand on the mechanism by which SA triggered patchoulol accumulation, we conducted an analysis of the transcription factors affected by SA treatment in *P. cablin*. Our selection of candidate genes focused on “*Pat_A04G047900*”, which demonstrated significant up-regulation in the transcriptome of the SA-treated pool. By conducting an evolutionary tree analysis, we discovered that *Pat_A04G047900*’s encoded protein closely resembled the *salvia*’s WRKY71 protein. As a result, we named it PatWRKY71 (Fig. 4A). To assess *PatWRKY71*’s transcription levels, we performed qRT-PCR and observed a noticeable increase following SA treatment (Fig. 4B). Additionally, we investigated the expression pattern of *PatWRKY71* by analyzing its transcript levels in stem, root, and leaf tissue. Notably, *PatWRKY71* was found to be expressed in all detected tissues, with particularly high levels detected in the root tissue (Fig. 4C). Thirdly, in order to study the subcellular localization of PatWRKY71, we constructed *UBQ10: GFP: PatWRKY71* and transiently expressed it in protoplasts alongside Nu-mCherry as a marker. Upon observation through confocal microscopy, it was found that the GFP fluorescence co-localized with the Nu marker, indicating that PatWRKY71 was localized in the nucleus (Fig. 4D).

### PatWRKY71 can bind to the promoter of *PatPTS* and increased its expression

**Fig. 5:**
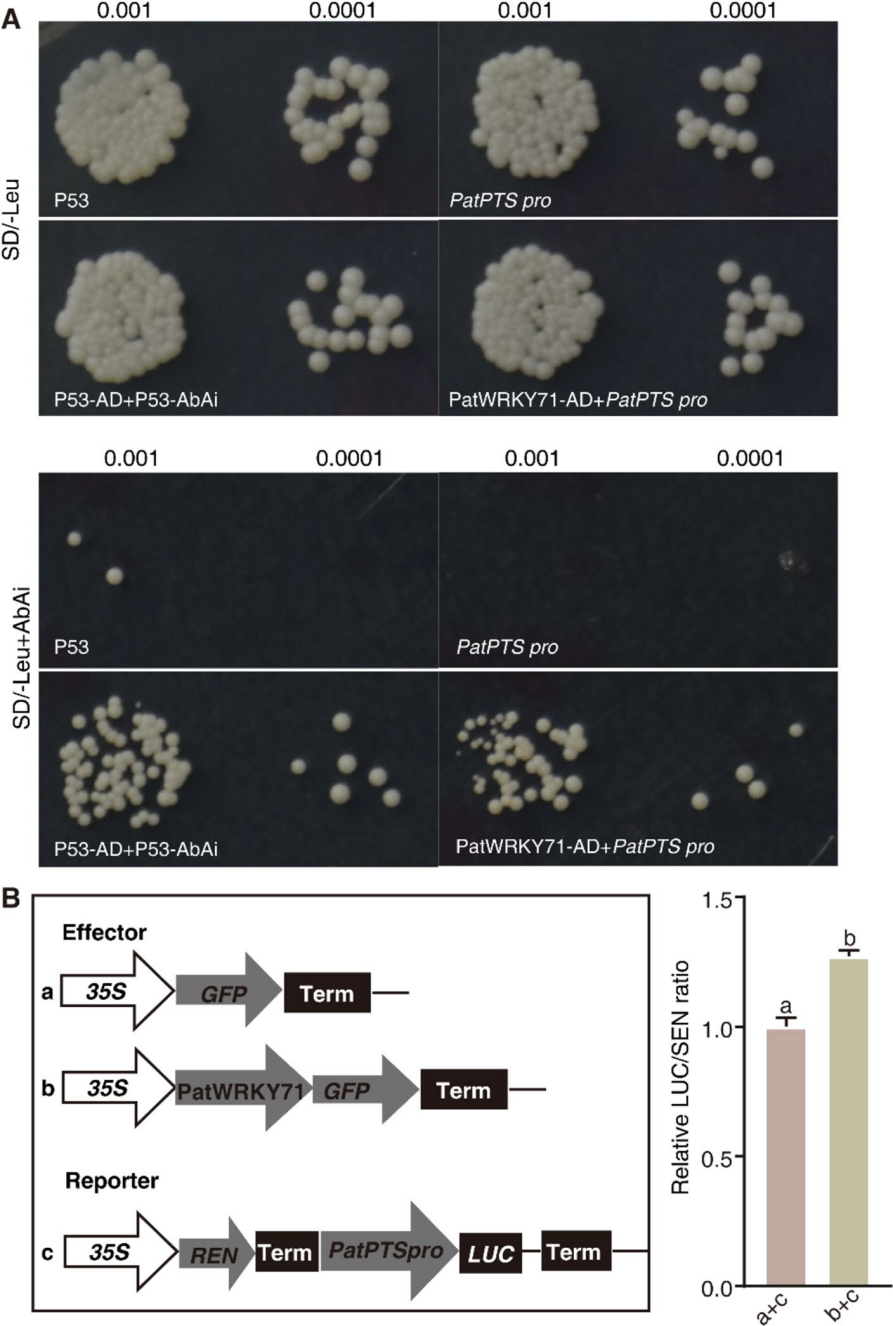
PatWRKY71 bound to the promoter of *PatPTS*. **A** Y1H assay confirmed the interaction between PatWRKY71 and *PatPTS* promoter. p53-pAbAi and p53-pGADT7 were used as potive control. The transformed yeast was grown on SD/-Leu with or without 200 ng/ml AbAi media for 3 d and images were recorded. **B** Dual luciferase(dual-LUC) reporter assay indicated that PatWRKY71 activated the transcription of *PatPTS* in *N. benthamiana* leaves. The effector plasmid was *35S: PatWRKY71*. Reporter plasmids contained *35S: REN- PatPTS pro: LUC*. Negative a control was *35S: GFP*. The relative LUC/REN ratio represents the relative activity of the *PatPTS* promoter. Different letters indicated significant differences from the control at *p* < 0.05 according to Student’s *t*-test (*p* < 0.05). Means ± SD (*n* = 3) are shown

WRKY proteins are specialized transcription factors found exclusively in plants, able to specifically bind to the W box of their target genes [25]. In our subject paper, we utilized a yeast one-hybrid experiment to determine that PatWRKY71 has the ability to bind to the promoter of *PatPTS* (Fig. 5A). This finding was then verified through a dual-luciferase (LUC) assay. Specifically, the effector plasmid, which featured PatWRKY71, was drove by the CaMV35S promoter (*35S: PatWRKY71*). In conjunction with this, the reporter plasmids were composed of LUC, driven by the *PatPTS* promoter (*PatPTS pro: LUC*), and renilla (REN) luciferase, driven by the CaMV35S promoter. We co-expressed the effector plasmid and reporter plasmid in tobacco leaves and our findings revealed that PatWRKY71 acts as a transcriptional enhancer of *PatPTS* expression, as its expression resulted in increased *PatPTS* expression compared to the control group of GFP (Fig. 5B).

### Virus-induced silencing of *PatWRKY71* decreased patchoulol biosynthesis

**Fig. 6:**
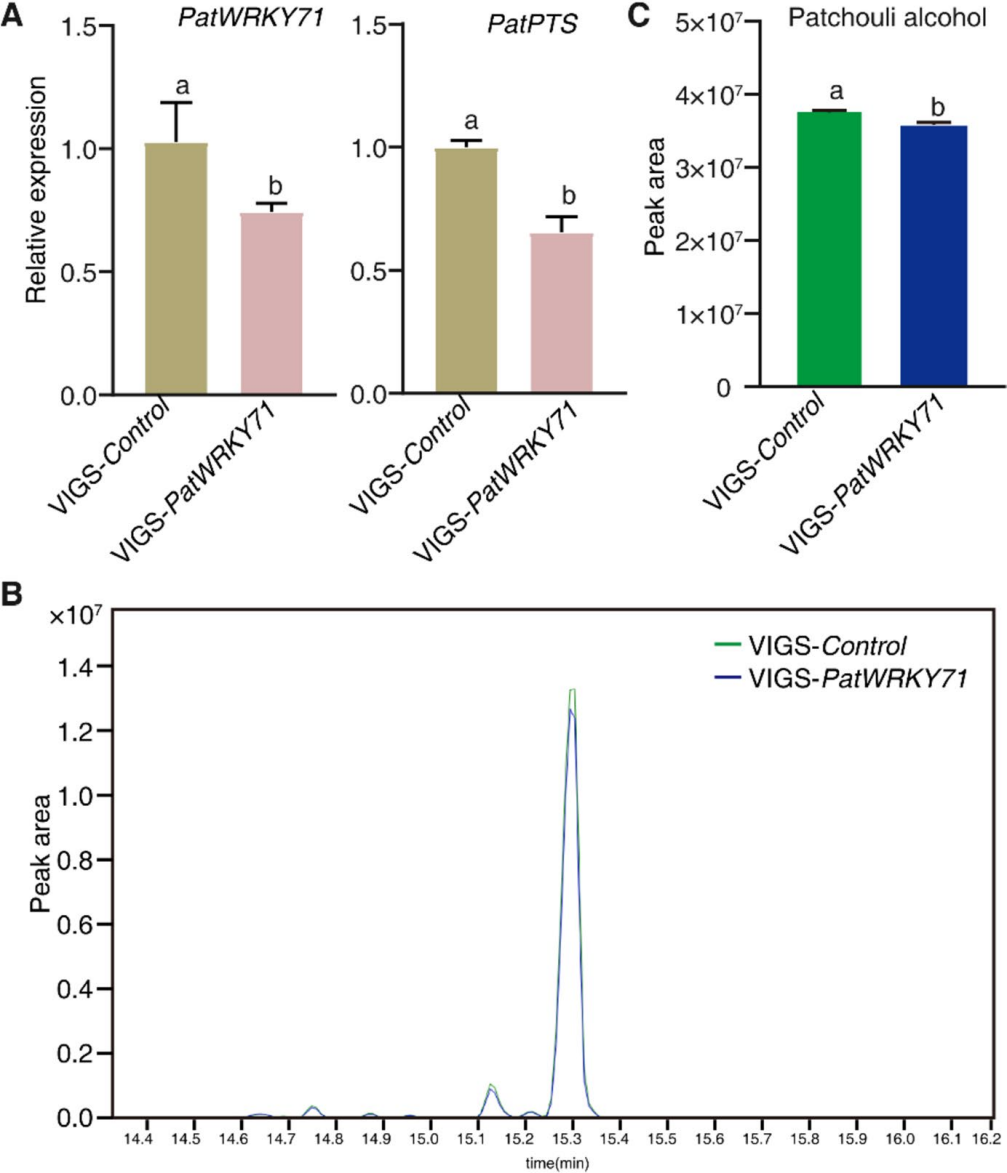
virus-induced silencing of PatWRKY71 decreased patchoulol production. **A** AThe relative expression level of *PatWRKY71* and *PatPTS* after *PatWRKY71*-VIGS. **B** The chromatogram of patchoulol after *PatWRKY71*-VIGS. **C** Detection of patchoulol in empty vector control (Control-VIGS) and *PatWRKY71*-VIGS leaves. Different letters indicated significant differences from the control at *p* < 0.05 according to Student’s *t*-test (*p* < 0.05). Means ± SD (*n* = 3) are shown

To illustrate the role of *PatWRKY71* in patchoulol biosynthesis, we performed a VIGS experiment on the *PatWRKY71* gene. After 14 days of silencing, leaf samples were collected for determining target genes expression and the content of patchoulol. Using qRT-PCR, we found the expression of *PatWRKY71* and *PatPTS* were decreased significantly (Fig. 6A). GC–MS analysis demonstrated a significant reduction in patchoulol production in the *PatWRKY71*-VIGS group, which was consistent with the results of qRT-PCR (Fig. 6B, C).

### Heterologous expression of *PatWRKY71* increased salt sensitivity in* A. thaliana*

**Fig. 7:**
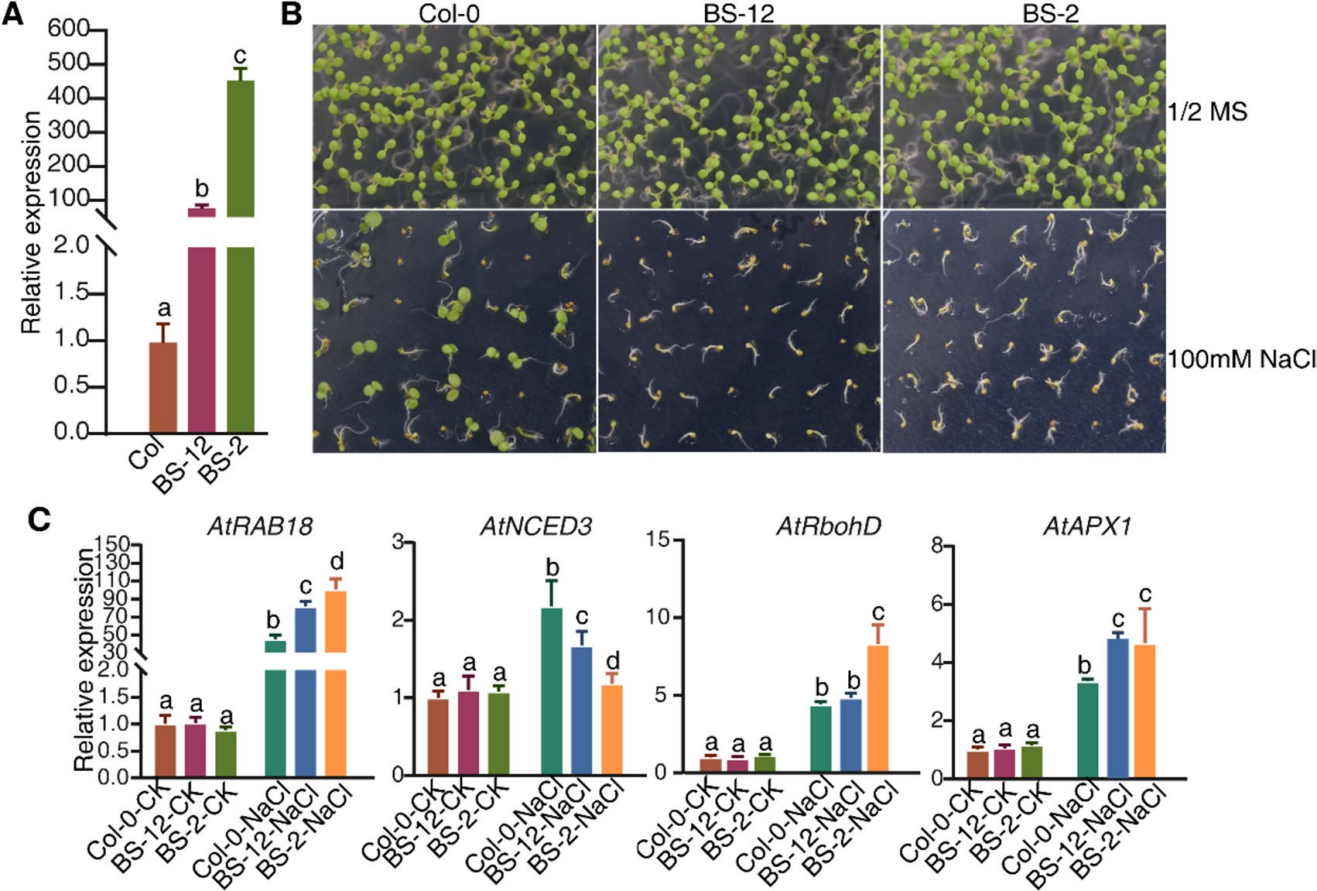
Heterologous expression of *PatWRKY71* increased salt sensitivity in Arabidopsis. **A** Detection of expression of *PatWRKY71*. The *AtACT2* expression level was used as the internal control. Gene expression in Col samples was set as 1 for normalization. Different letters represent a significant difference from the Col-CK using Student’s *t*-test (*p* < 0.05). **B**, **C** Surface-sterilized seeds were sown on 1/2 × MS with or without 100 μM NaCl and grown horizontally in greenhouse at 22 °C a 16-h light / 8-h dark conditions. Photos were recorded after 2 weeks’ incubation (**B**). Seedlings were collected for measuring the expression of target genes (**C**). The *AtACT2* expression level was used as the internal control. Gene expression in Col-CK samples was set as 1 for normalization. Different letters represent a significant difference from the Col-CK using Student’s *t*-test (*p* < 0.05)

To determine whether *PatWRKY71* was involved in plant defense regulation as well as patchoulol production, we conducted heterologous overexpression of *PatWRKY71* in *A. thaliana*. Firstly, we measured the transcription level of *PatWRKY71* in the homozygous overexpressing *A. thaliana* lines (BS-12 and BS-2). The result showed that the expression level of *PatWRKY71* in BS-12 and BS-2 was significantly higher than that in the wild type Col-0 plants (Fig. 7A). Secondly, we performed a plate assay to examine salt tolerance of wild-type Col-0 and BS-12 and BS-2 plants. Seeds were surface-sterilized and then sown on 1/2 × MS plates containing 100 μM NaCl. After incubating for 10 days, photos were recorded and we found BS-12 and BS-2 were more sensitive to NaCl treatment than Col-0 (Fig. 7B). We also collected salt-treated samples for qRT-PCR assay and found the relative transcription levels of salt pathway-related genes (*AtRAB18*, *AtNCED3*) and ROS-responsive genes (*AtRbohD*, *AtAPX1*) were higher in salt-treated BS-12 and BS-2 than that in the wild type Col-0 (Fig. 7C).

### Heterologous expression of *PatWRKY71* increased Cd sensitivity in* Arabidopsis*

**Fig. 8:**
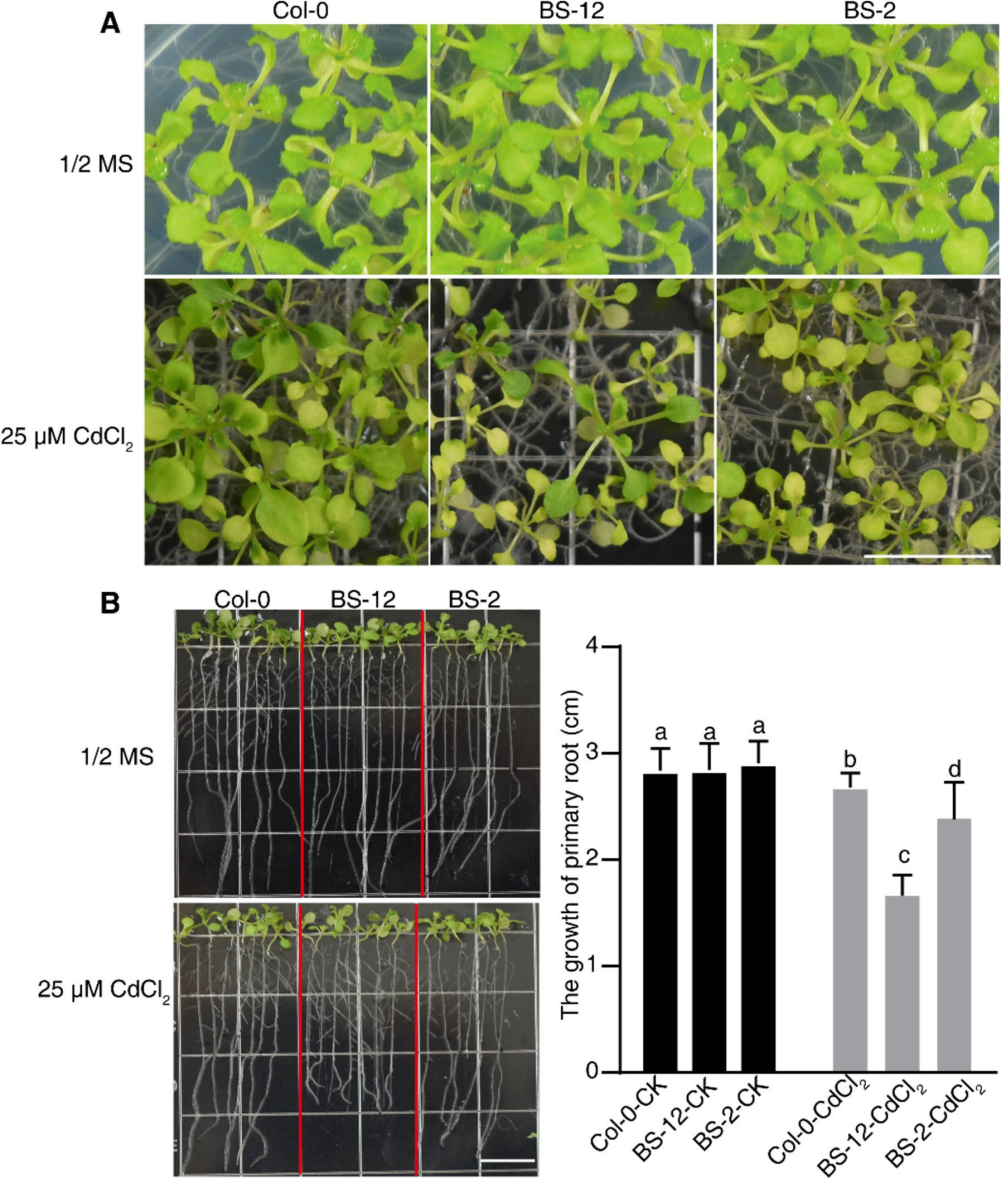
Heterologous expression of *PatWRKY71* increased Cd sensitivity in Arabidopsis. Surface-sterilized seeds were sown on 1/2 × MS containing 25 μM CdCl_2_ and grown horizontally for 3 weeks’ incubation and recorded phenotype (**A**). For root growth experiment, seedlings grown vertically on 1/2 × MS for 7 days and then transferred to 1/2 × MS containing 25 μM CdCl_2_ and the length of root were measured (**B**)

To determine if the overexpression of *PatWRKY71* in *A. thaliana* could enhance plant defense against cadmium exposure, we conducted an experiment. Surface-sterilized seeds were planted on 1/2 × MS plates with or without 25 μM CdCl_2_ for a duration of 2 weeks. We documented photos and observed that BS-12 and BS-2 were smaller than Col-0 following Cd treatment (Fig. 8A).

Next, we also conducted a root growth assay. We sowed surface-sterilized seeds on 1/2 × MS plates and allowed them to grow vertically for 7 days. We then transferred the seedlings to 1/2 × MS plates with or without 25 μM CdCl_2_ and measured the length of root elongation. Our results showed that there was no difference in root length between BS-12 or BS-2 and Col-0 under control conditions. However, we did observe a significant difference in root length between BS-12 and BS-2 versus Col-0 under Cd treatment, with the former two being shorter (Fig. 8B).

**Fig. 9:**
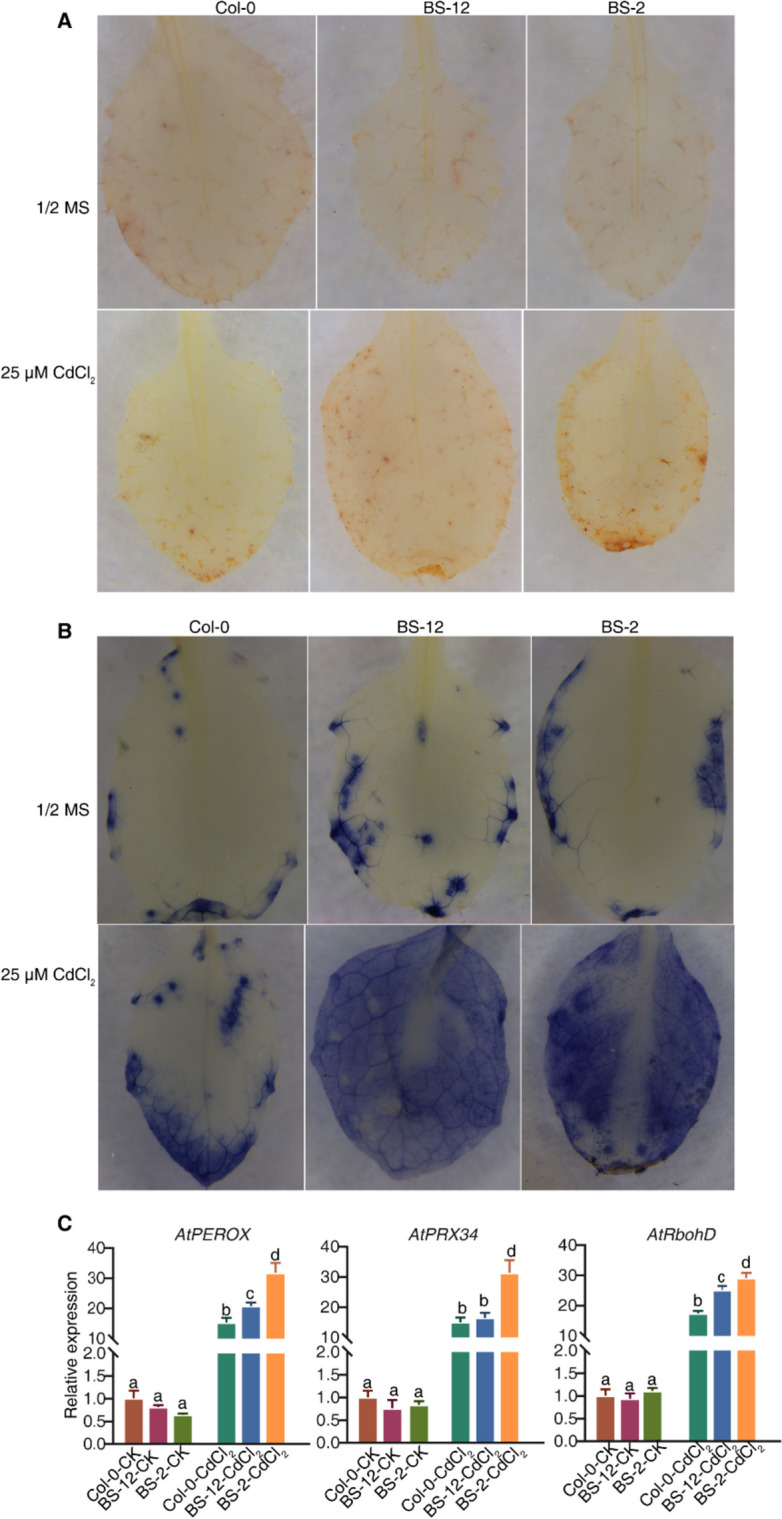
Over- expression of *PatWRKY71* induced ROS production under Cd stress in Arabidopsis**.** Surface-sterilized seeds were sown on 1/2 × MS containing 25 μM CdCl_2_ and grown horizontally for 3 weeks’ incubation and leaves were collected for H_2_O_2_ detection using DAB staining (**A**), O_2_.^.−^ detection using NBT staining (**B**) and gene expression using qRT-PCR (**C**). Gene expression in Col-CK samples was set as 1 for normalization. Different letters represent a significant difference from the Col-CK using Student’s *t*-test (*p* < 0.05)

Additionally, we assessed the levels of H_2_O_2_ and O_2_^·−^ in Col-0 and BS-12 or BS-2 plants when subjected to Cd treatment. To monitor H_2_O_2_, the third and fourth true leaves were stained with 3,39-diaminobenzidine tetrahydrochloride (DAB), while nitroblue tetrazolium (NBT) was used as a histochemical reagent for O_2_^·−^. Our findings showed that BS-12 or BS-2 plants displayed a significant increase in the accumulation of H_2_O_2_ and O_2_^·−^ when exposed to Cd treatment (Fig. 9A, B).

Finally, we collected samples for a qRT-PCR assay and discovered that the relative transcription levels of ROS-responsive genes (*AtPEROX*, *AtPRX34*, *AtRbohD*) were higher in Cd-treated BS-12 and BS-2 compared to the wild type Col-0 (Fig. 9C).

## Discussion

Plant hormones not only control plant growth and development but also act as regulators of plant secondary metabolism [26]. Patchouli alcohol, a sesquiterpenoid, is the main medicine produced from *P. cablin* and has drawn significant attention due to its high market demand [27]. As a result, an increasing number of researchers have investigated the role of MeJA in patchouli alcohol production [3, 10, 11, 28]. The role of salicylic acid (SA) in the molecular synthesis mechanism of patchouli alcohol has been unclear. However, our study shows that SA can significantly improve patchouli alcohol production, as detected by GC–MS.

To date, the nonexpressor of PR genes (NPRs), including NPR1, NPR3, and NPR4, have been identified as SA receptors that bind SA directly. These receptors are essential for regulating the expression of PR genes [29]. Although NPRs lack DNA binding domains, they participate in signal transduction by interacting with transcription factors like TGAs [30]. NPR1, in particular, has been implicated in regulating plant responses by directly upregulating the expression of *WRKY* transcription factor genes [31]. Yu et al. discovered that W-box is present in the promoter of NPR1, and it can be identified by SA-induced WRKY proteins in *Arabidopsis* [32]. Additionally, SA treatment can induce the expression of *AtWRKY18*, *AtWRKY58*, and *AtWRKY70*, which were important regulatory nodes in the systemic acquired resistance (SAR) pathways [33]. This indicated that WRKY transcription factors played a critical role in the SA signaling pathway. In order to investigate how SA enhances patchouli alcohol production, we conducted a transcriptome analysis of *P. cablin* plants treated with SA, and identified differentially expressed WRKY genes. Among these, *PatWRKY71* was found to be significantly induced in the leaves after SA spraying. Further experiments using yeast one-hybrid and dual-luciferase assays revealed that PatWRKY71 can bind to the promoter region of *PatPTS* and up-regulate its expression. Silencing *PatWRKY71* resulted in down-regulation of both *PatWRKY71* and *PatPTS* expression, ultimately leading to a reduction in the content of patchouli alcohol. These findings suggest that SA may improve patchouli alcohol production by activating the transcription factor *PatWRKY71*, which can bind to *PatPTS* and up-regulate its expression.

Salicylic acid is a compound that is commonly found in plants and is utilized in their defense against biotic or abiotic stressors [34, 35]. Research has shown that SA can mitigate the effects of salt stress caused by NaCl by promoting the accumulation of proline [36]. Additionally, Yang et al. discovered that SA is a key factor in enhancing salt tolerance through its interaction with sphingolipids, which are primary metabolites in plants [37]. Many studies have shown that WRKY transcription factors are linked to plant resistance against salt stress. In *Populus euphratica*, five *PeWRKY* genes were found to have altered expression under salt stress [38]. Interestingly, overexpression of *ZmWRKY114* in rice led to increased sensitivity to salt stress [39], while overexpression of *SlWRKY8* in tomato resulted in improved salt stress tolerance [40]. Furthermore, SA has been shown to play a crucial role in regulating plant responses to Cd toxicity [41].

Research has revealed that SA has the ability to improve Cd tolerance in numerous plant species. However, it's important to note that pre-treating castor bean seedlings with SA actually intensified Cd damage [35]. To further examine whether *PatWRKY71* contributes to plant defense against abiotic stresses, we conducted a study where we heterologous expressed* PatWRKY71* in *Arabidopsis*. The results showed that plants overexpressing *PatWRKY71* exhibited heightened sensitivity to both salt and Cd, indicating that *PatWRKY71* has a negative impact on plant resistance. In plants overexpressing *PatWRKY71*, there was a higher level of ROS production under salt or Cd stress compared to the wild-type, indicating that *PatWRKY71* worsened oxidative damage caused by salt or Cd treatment. This aligns with existing literature, which shows that Cd increases ROS production by altering the activity of the NADPH oxidase [42, 43].

## Conclusion

Through our study, we discovered that SA has the potential to enhance patchoulol production. Additionally, we identified a downstream transcription factor, PatWRKY71, through transcriptome analysis of *P. cablin* that had been treated with SA. PatWRKY71 was a homologous protein of *Arabidopsis* WRKY71 and was primarily expressed in roots. Our findings also revealed that PatWRKY71 was located in the nucleus and can bind to and promote the promoter activity of *PatPTS*, as demonstrated by Y1H system and dual-LUC assays. In the same vein, the expression level of the *PatPTS* gene and the accumulation of patchoulol experienced a significant drop when the *PatWRKY71* gene underwent virus-induced gene silencing (VIGS) in *P. cablin* leaves. The heterologous expression of *PatWRKY71* leaded to an increase in salt and Cd sensitivity in *Arabidopsis*, accompanied by an outbreak of ROS. Our research has revealed the regulatory role of PatWRKY71 in patchoulol biosynthesis and plant defense response.

### Supplementary Information

**Additional file 1: Supplemental Table 1. **The list of primers used in this study.

## Data Availability

The datasets used and/or analysed during the current study available from the corresponding author on reasonable request.
