# Supplementary material for: PatWRKY71 transcription factor regulates patchoulol biosynthesis and plant defense response
Source: BMC Plant Biol. 2024 Jan 2;24:8. doi: 10.1186/s12870-023-04660-7 (PMC10759419; doi:10.1186/s12870-023-04660-7)
Supplement: Supplementary file 1 — Additional file 1: Supplemental Table 1. The list of primers used in this study. [file 12870_2023_4660_MOESM1_ESM.docx]

| **Supplemental Table 1.** The list of primers used in this study | | |
| --- | --- | --- |
| **Purpose** | **Name** | **Sequence (5’ to3’)** |
| **Primers for PatWRKY71 cloning**  **and localization** |  |  |
| PatWRKY71 | LP | GACGAGCTGTACAAGAGGCCTATGTCGGAAGATTACACCG |
|  | RP | GTCGTCCTTGTAGTCAGGCCTGGCCTCCTCTTTAGGGTAAACG |
| **Primers for VIGS**  PatWRKY71 | LP | TAAGGTTACCGAATTCATGATGAGAACTCCAAGAAAGAATG |
|  | RP | ATGCCCGGGCCTCGAGAGTCGGCGAACTGGTGGAA |
| **Primers for Dual-LUC assay** | | |
| PatWRKY71 | *FP* | GACGAGCTGTACAAGAGGCCTATGTCGGAAGATTACACCG |
|  | *RP* | GTCGTCCTTGTAGTCAGGCCTGGCCTCCTCTTTAGGGTAAACG |
| *PatPTSpro* | *FP* | TTCCTGCAGCCCGGGGGATCCATGAGATCCACATAGATCAATCACTTTA |
|  | *RP* | CGCTCTAGAACTAGTGGATCCTACGGCTAGCTCGACTGTGACTG |
| **Primers for Y1H assay** |  |  |
| *PatWRKY71* | *FP* | GCCATGGAGGCCAGTGAATTCATGTCGGAAGATTACACCG |
|  | *RP* | CAGCTCGAGCTCGATGGATCCCGGCCTCCTCTTTAGGGTAAACG |
| *PatPTSpro* | *FP* | AATGATGAATTGAAAAGCTTATGAGATCCACATAGATCAATCACTTTA |
|  | *RP* | G TCGACAGATCCCCGGGTACCTACGGCTAGCTCGACTGTGACTG |
| **Gene expression detection primers** |  |  |
| *Pat18S* | *FP* | TCGCCGTTCGGACCAAATAA |
|  | *RP* | CGATGGTTCACGGGATTCTGC |
| *PatWRKY71* | *FP* | ATGTAAAGCGAAGAAGAAAGGGGAG |
|  | *RP* | TAGCTTCTTGGATAAGGGCTGTTCT |
| *PatPTS* | *FP* | TGGGTGCTGCTTCTCGTCCTC |
|  | *RP* | TGCGTTGTGGACTTGTTTCG |
| *AtACT2* | *FP* | ATGTGGATTGCCAAGGCTGA |
|  | *RP* | CGGCGATAACAGCTCCTCTT |
| *AtRAB18* | *FP* | AGGAGAAGTTGCCAGGTCAT |
|  | *RP* | CGTAGCCACCAGCATCATATC |
| *AtNCED3* | *FP* | ACAAGAACAAGGTCGCAAGATT |
|  | *RP* | ATTGAGTCTGGTGGAGTCATACA |
| *AtPRX34* | *FP* | CCTACACTCAACACTACTTACCT |
|  | *RP* | CTCTTGGTCGCTCTGGATAA |
| *AtRbohD* | *FP* | CGACAGCGACATTGAGAACA |
|  | *RP* | AGTTCTGTCTTCAACCACCTG |
| *AtPEROX* | *FP* | GACTATCATCAACAGCGGCTAG |
|  | *RP* | TGGCGAAGAGTGAAGTGAGT |
| *AtAPX1* | *FP* | CTGACAAAGCACTATTGGACGACCC |
|  | *RP* | TCAGAAAGCTTCATGTGGGCCTCAG |
